# Whole genome analysis of a livestock-associated methicillin-resistant *Staphylococcus aureus *ST398 isolate from a case of human endocarditis

**DOI:** 10.1186/1471-2164-11-376

**Published:** 2010-06-14

**Authors:** Maarten J Schijffelen, CH Edwin Boel, Jos AG van Strijp, Ad C Fluit

**Affiliations:** 1 Department of Medical Microbiology, University Medical Centre Utrecht, Heidelberglaan 100, 3508 GA, Utrecht, the Netherlands

## Abstract

**Background:**

Recently, a new livestock-associated methicillin-resistant *Staphylococcus aureus *(MRSA) Sequence Type 398 (ST398) isolate has emerged worldwide. Although there have been reports of invasive disease in humans, MRSA ST398 colonization is much more common in livestock and demonstrates especially high prevalence rates in pigs and calves. The aim of this study was to compare the genome sequence of an ST398 MRSA isolate with other *S. aureus *genomes in order to identify genetic traits that may explain the success of this particular lineage. Therefore, we determined the whole genome sequence of S0385, an MRSA ST398 isolate from a human case of endocarditis.

**Results:**

The entire genome sequence of S0385 demonstrated considerable accessory genome content differences relative to other *S. aureus *genomes. Several mobile genetic elements that confer antibiotic resistance were identified, including a novel composite of an type V (5C2&5) Staphylococcal Chromosome Cassette *mec *(SCC*mec*) with distinct joining (J) regions. The presence of multiple integrative conjugative elements combined with the absence of a type I restriction and modification system on one of the two νSa islands, could enhance horizontal gene transfer in this strain. The ST398 MRSA isolate carries a unique pathogenicity island which encodes homologues of two excreted virulence factors; staphylococcal complement inhibitor (SCIN) and von Willebrand factor-binding protein (vWbp). However, several virulence factors such as enterotoxins and phage encoded toxins, including Panton-Valentine leukocidin (PVL), were not identified in this isolate.

**Conclusions:**

Until now MRSA ST398 isolates did not cause frequent invasive disease in humans, which may be due to the absence of several common virulence factors. However, the proposed enhanced ability of these isolates to acquire mobile elements may lead to the rapid acquisition of determinants which contribute to virulence in human infections.

## Background

*Staphylococcus aureus *is an important pathogen that can cause a wide variety of hospital- and community-acquired infections. In addition, it is also part of the normal flora in 20-40% of adult humans [1]. In hospitals throughout the world, antibiotic pressure combined with the adaptive genetic capacity of *S. aureus *has resulted in the development of lineages with resistance to multiple antimicrobial drugs, including methicillin. Hospital-associated methicillin-resistant *S. aureus *(HA-MRSA) has become a major source of nosocomial infections and is associated with increased morbidity and mortality [2]. However, in the past decade, MRSA has increasingly caused infections and outbreaks outside the hospital setting, particularly among healthy people who lack the known risk factors for MRSA acquisition. These isolates are referred to as community-associated MRSA (CA-MRSA) and are genetically distinct from HA-MRSA. Considerable genetic heterogeneity has been observed among CA-MRSA strains, which contrasts with the presence of a few pandemic clones of HA-MRSA [3]. In 2003, a new MRSA lineage, Sequence Type 398 (ST398), emerged in the community [4,5]. ST398 isolates can cause several acute infections including benign skin and soft tissue infections, respiratory tract infections, as well as serious life-threatening conditions such as bacteremia and endocarditis [6-8]. Recent studies have shown that a major risk factor in ST398 human infections is a professional relationship with livestock farming, especially with pigs and calves [9-12]. Indeed, MRSA ST398 colonization of these animals is quite common, and overall prevalence rates for pigs are as high as 49% [13]. This is the first time that such a large MRSA reservoir was identified outside of a hospital environment. The identification of livestock in MRSA ST398 transmission is of considerable concern, especially since livestock is thought to be the source of several emerging antimicrobial-resistant bacteria in the community. Currently, MRSA ST398 has been identified throughout the world, including countries in Europe, North America and Asia [14-18]. Thus, MRSA ST398 represents a distinct lineage that has rapidly spread worldwide and is mainly associated with animal colonization in livestock farming. However, this isolate is also able to colonize and cause invasive disease in humans. Therefore, the entire genome sequence of an ST398 isolate was determined and compared with other *S. aureus *genomes, in order to identify genetic traits that may explain the success of this global lineage.

## Results

The MRSA ST398 genome consists of a circular chromosome of 2,872,582 bp, as well as 3 circular plasmids. The size of the chromosome is comparable to that of other sequenced *S. aureus *strains. We identified 2,699 ORFs, 6 ribosomal RNA operons, and 24 mobile genetic elements, including 12 insertion sequence (IS) elements. The general features of the genome are summarized in Table 1. In total, 37 predicted genes had no significant homologues in public databases. The accessory component of the genome is mainly represented within 12 mobile genetic elements on the chromosome and in the three plasmids. Most of these genetic elements carry virulence and resistance genes involved in colonization and pathogenesis, and are likely acquired horizontally as indicated by integration at specific loci and/or the presence of recombinase genes, flanking direct repeats, as well as GC content. As observed in other *S. aureus *genomes, the majority of unique genes are encoded on allotypes of known mobile genetic elements.

**Table 1:** General characteristics of the *S. aureus *S0385 genome

	Chromosome	pS0385-1	pS0385-2	pS0385-3
Size (base pairs)	2,872,582	5,246	4,381	3,158
G+C content (%)	32.9%	30.7%	31.6%	29.0%
Protein-coding genes	2699	4	6	2
Coding (%)	83.5%	77.5%	78.1%	60.7%
Transfer RNA genes	59	0	0	0
Ribosomal RNA operons	6	0	0	0
Insertion sequences	12	1	0	0
Mobile genetic elements	12	0	0	0
Transposons	3			
Prophages	2			
SCC*mec*	1			
SaPI	1			
νSa islands	2			
ICE	3			

**Figure 1:**
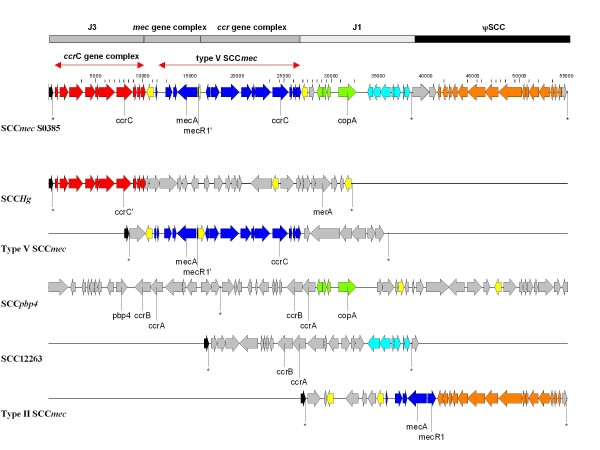
**Structure and comparative analysis of SCC*mec *element of S0385**. The SCC*mec *element of S0385 contains a *mec *and *ccr *gene complex with a nearly identical structure compared to type V SCC*mec*, and two variable non-essential regions that include J1 and J3, the latter which contains a second solitary *ccrC *gene complex [19]. A pseudo-SCC element (ψSCC) is integrated upstream in tandem with the SCC*mec *element. Asterisks indicate the three direct repeat containing integration site sequences. The S0385 SCC*mec *element appears to be composed of gene clusters that have been previously described for other SCC elements. Shown is an alignment of the S0385 SCC*mec *element with the other SCC elements (i.e. *S. aureus *SCC*Hg*, strain85/2082 and type V SCC*mec*, strain WIS; *S. epidermidis *SCC*pbp4*, strain ATCC12228; *S. hominis *SCC12263, strain GIFU12263 and *S. aureus *type II SCC*mec*, strain JCSC6826). Arrows represent ORFs and their direction of transcription. The homologous clusters of ORFs are indicated with similar colors. Red: solitary *ccrC *gene complex; blue: SCC*mec *type V *mec *and *ccrC *gene complex; green: cluster containing *copA *gene; aqua: metallo-hydrolase gene complex; orange: integrative conjugative element; yellow: IS431; black: *orfX*; grey: ORFs with no homology to the other SCC elements shown.

A 38 kb type V (5C2&5) Staphylococcal Cassette Chromosome *mec *(SCC*mec*), which contains the *mecA *gene that confers methicillin resistance, is inserted at the characteristic chromosomal integration site (3'-end of the *orfX *gene) of MRSA S0385. Type V SCC*mec *is characterized by the presence of a *ccrC *gene in the ccr gene complex, which encodes a site-specific recombinase that is required for excision and integration of the SCC*mec *element that is flanked by direct repeats containing the integration site sequence [19]. The S0385 SCC*mec *element appears to be composed of gene clusters that have been previously described for other SCC elements (Figure 1 and Additional file 1: Table S1). The variable non-essential joining 3 (J3) region harbors a second intact *ccrC *gene complex directly downstream of *orfX*. This region is similar to the *ccrC *complex in SCC*Hg *[20]. Although SCC*Hg *contains a truncated *ccrC *gene, it can still be mobilized due to the presence of flanking integration site sequences, while the neighboring type III SCC*mec *element may provide a recombinase. Similar alternative excision has been described for another MRSA strain carrying a type IV SCC*mec *with additional integration site sequences [21]. However, type V SCC*mec *in MRSA S0385 should be considered a composite element because it lacks the additional integration site sequence that is required for alternative excision of the two elements. In addition, within region J1, the SCC*mec *element of S0385 carries a *copA *gene that may confer copper resistance. This gene was previously found only in the SCC*pbp4 *element of *Staphylococcus epidermidis *ATCC 1228 [22]. Finally, a truncated metallo-hydrolase gene complex, similar to *Staphylococcus hominis *SCC12263 was also identified in this isolate.

Type V (5C2&5) SCC*mec *elements with a *ccrC *gene containing J3 region have recently been found in a MRSA ST59 isolate [GenBank: AB462393] and a methicillin-resistant *Staphylococcus pseudintermedius *isolate [GenBank: FJ544922]. Each of these isolates has an integration site sequence at *orfX *which is identical to that of the SCC*mec *element in S0385. However, they possess numerous SNPs in the intergenic region upstream of the first IS*431 *element and in the final two ORFs of the *ccr*C complex. Furthermore, the content of the J1 region is unique for all three elements and a transposon is also integrated into the SCC*mec *of MRSA ST59.

Immediately downstream of SCC*mec *is a second 16.5 kb pseudo-SCC element that is integrated into *orfX *(Figure 1 and Additional file 1: Table S1). Although it is flanked by SCC-specific integration site sequences, this second element lacks any *ccr *genes suggesting that it depends on the SCC*mec *encoded recombinase for excision. The pseudo-SCC element carries 20 ORFs of which three show no homology to any known genes and 17 represent a gene cluster with homology to integrative conjugative elements (discussed in more detail below). This gene cluster is also present in the *S. aureus *genomes of COL, USA300 FR3757 and MRSA252, but is always integrated at integration sites not related to an SCC element. Recently, one example of this 17 gene cluster has been described in association with an SCC element. It was integrated into the J1 region of a type II SCC*mec *from isolate JCSC6826. However, the gene cluster was integrated without SCC-specific integration site sequences and lacks the additional three ORFs that flank the pseudo-SCC element in S0385.

Two nearly identical bacteriophages of approximately 47 kb, φSa6S0385 and φSa2S0385, have integrated into the chromosome at the lipase gene and a hypothetical protein, respectively. The attachment site and integrase of φSa6S0385 and φSa2S0385 were similar to those of the φCOL and φSa2 family, respectively. The two phages demonstrated considerable differences at the terminal ends, which include the integrase and endolysin genes. The domains that were common to both bacteriophages had a mosaic structure and consisted mainly of genes encoding phage regulatory, tail and capsule proteins. We were not able to identify any known virulence gene besides NWMN0280, which is a virulence associated gene previously identified in the phages of *S. aureus *Newman. The remarkable presence of two highly homologous phages has only been described once before in the *S. aureus *Newman strain [23].

Three transposons were integrated into the core chromosome. A transposon similar to Tn*916 *of *Enterococcus faecalis *carries the *tet*(M) tetracycline resistance element and a gene cluster required for conjugative transfer. The second transposon, Tn*552*, encodes an inducible β-lactamase and its regulator components. Both elements are commonly detected in tetracycline- and β-lactam-resistant staphylococci, respectively. A third transposon showed similarity with the Tn*7 *transposon family. Members of this family are usually found in Gram-negative bacteria. However, recently Tn*7*-like transposons have been reported in Gram-positive bacteria, such as *Bacillus cereus *and staphylococci [24]. It is unknown which function(s) are encoded by the Tn*7*-like transposon in S0385 as it lacks typical genes that are known to confer antibiotic resistance or enhance pathogenesis.

**Figure 2:**
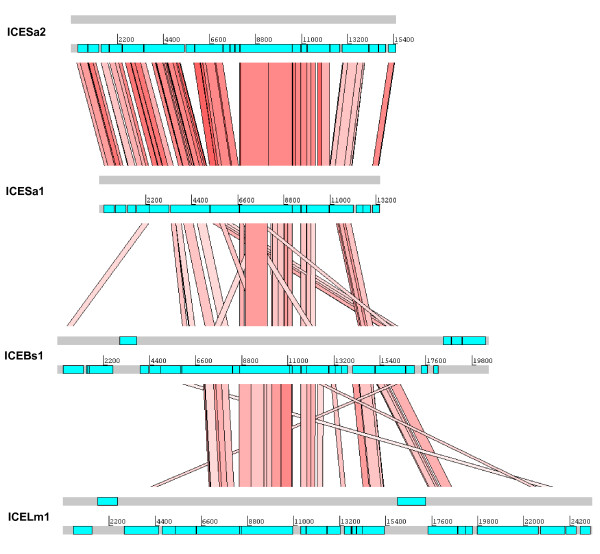
**Compairison of the integrative conjugative elements**. Amino acid matches from the six-frame translations (TBLASTX) of integrative conjugative elements of *S. aureus *S0385 (ICESa2 and ICESa1), *B. subtilis *(ICEBs1) [GenBank: AL009126 ] and *L. monocytogenes *(ICELm1) [GenBank: AARQ] dislayed using ACT http://www.sanger.ac.uk/software/ACT. ORFs are represented by coloured boxes. The red bars indicate TBLASTX matches between the elements. There are six proteins that are conserved among all ICEs (SAPIG0075, 0076, 0077 and 0079-0081), which include the FtsK/spoIII and replication initiation proteins.

Two nearly identical 15 kb putative mobile genetic elements were integrated into the genome. One was contained in the pseudo-SCC element while the other was integrated into the acyl-coenzyme A ligase gene. The integration appears to result in the duplication of 3 nucleotides. Furthermore, nearly identical elements were also integrated in the USA300 FPR3757, MRSA252 and COL genomes, as well as type II SCC*mec *from the isolate JCSC6833. The element contains 17 ORFs, which includes proteins with conserved domains related to amidase, ATPase, DNA translocase, a replication initiation factor and a gene with a frame-shift encoding an integrase. Frame-shifts among integrase genes have been previously reported [25]. These frame-shifts are reversible and are thought to contribute to the regulation of the transfer of the genetic element. Nine of the 17 proteins demonstrate similarity with those found in integrative and conjugative elements from *Bacillus subtilis *(ICEBs1) and *Listeria monocytogenes *(ICELm1) (Figure 2 and Additional file 1: Table S2). Indeed, the circularized form of the element was detected by PCR (data not shown) suggesting that this element can be excised from the chromosome and may be transferred by conjugation with subsequent integration into the recipient. We named these elements, ICESa1A and ICESa1B. A third similarly organized element containing a cluster of genes homologous to ICESa1 was integrated into a conserved hypothetical protein and named, ICESa2 (Figure 2 and Additional file 1: Table S2). However, we did not identify any known virulence genes in these three elements.

**Figure 3:**
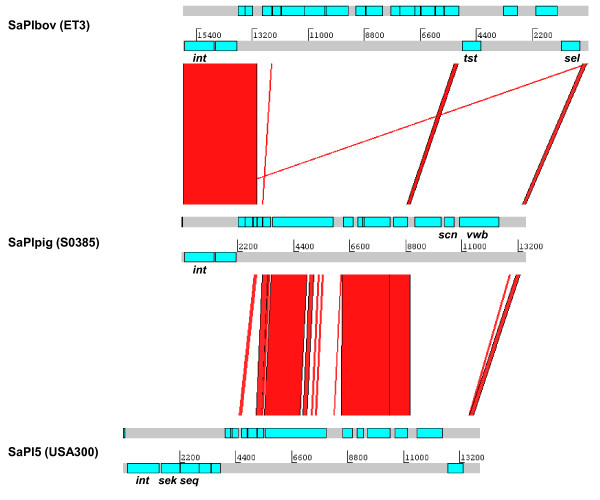
**Compairison of the SaPI**. Nucleotide matches of SaPIbov, SaPI-S0385 and SaPI5 of *S. aureus *ET3, S0385 and USA300 FPR3757, respectivly dislayed using ACT. ORFs are represented by coloured boxes. The red bars indicate BLASTN matches between the elements, which mainly concern regions encoding genes that are essential for SAPI replication. The unique region in SaPI-S0385 contains the *scn *and *vwb *genes.

A novel staphylococcal pathogenicity island (SaPI), designated as SaPI-S0385, was also identified. This SaPI was integrated into the same conserved 20 bp attachment site as SaPIbov (ET3) and composed at the 5'-end of sequences homologous to SaPIbov and SaPI5 (USA300 FPR3757). These sequences encode genes involved in transcription, replication and packaging (Figure 3 and Additional file 1: Table S3). A unique region at the 3'-end of SaPI-S0385 was identified to encode two putative extracellular proteins with similarity to staphylococcal complement inhibitor (SCIN) and von Willebrand factor-binding protein (vWbp), respectively. Both proteins also have a conserved homologue in the core genome of S0385. The other genes identified on SaPI-S0385 were a putative primase and hypothetical phage related genes.

Novel allelic variants of νSaα and νSaβ islands were also identified. Both of these νSa islands are present in all *S. aureus *genomes and each is integrated at a conserved position in the chromosome. There are considerable differences between the strains with respect to the genetic content of the νSa islands. However, a key element that has been observed in each νSa island is a type I restriction-modification (R-M) system. The presence of two R-M systems effectively prevents horizontal gene transfer into the genome and limits DNA transfer between *S. aureus *isolates to those of the same lineage [26]. Although both types of νSa islands are present in the S0385 genome, only νSaα harbors a type I R-M system, which has a unique *hsdS *gene that encodes the S subunit which determines the sequence specificity of type I R-M systems. The νSaα island also contains a staphylococcal superantigen-like (SSL) family of exotoxins as well as a family of lipoproteins. The proteins of the SLL family may play a role in innate immunity evasion, as SSL5 inhibits P-selectin-mediated neutrophil rolling and SSL7 binds IgA and complement factor C5 [27,28]. To our knowledge, a function for the family of lipoproteins has not yet been described. The νSaβ island harbors a hyaluronidase gene cluster that was also present in the bovine derived *S. aureus *strain, ET3. This cluster encodes a variant of the hyaluronidase encoded on the core genome of *S. aureus*. Hyaluronidase is a proposed virulence factor that is capable of degrading hyaluronic acid, a major component of the extracellular matrix in connective tissue, and thereby contributes to subcutaneous bacterial spread from the initial site of infection. Alternatively, the degradation products of hyaluronic acid may serve as a supply of nutrients for the organism [29,30]. The νSaβ island lacks enterotoxins, serine proteases, lantibiotic biosynthesis proteins and LukE/D hemolysins.

The genome of S0385 contained three plasmids. A tetracycline resistance determinant *tet*(K) is carried on plasmid pS0385-1, which is highly similar to pUSA02 from *S. aureus *USA300 FRP3757. A second plasmid carries a *str *gene which confers streptomycin resistance. Finally, a small cryptic plasmid was also identified (Table 1).

## Discussion

Analysis of the genome of *S. aureus *strain S0385, a member of the livestock-associated MRSA ST398 lineage, showed considerable differences relative to other *S. aureus *genome sequences. The majority of these differences were identified in unique or novel allotypes of mobile genetic elements. This finding stresses the importance of horizontal gene transfer in bacterial genome remodeling, as previously reported for other sequenced *S. aureus *genomes. Many of these mobile genetic elements harbor determinants for virulence and antimicrobial resistance which may allow the bacterium to adapt to new niches. It should be noted that some mobile elements such as SaPI-S0385, ICESa1, ICESa2, and phageSa6 do not appear to be present in the majority of 6 *spa*-types of MRSA ST398, as determined by PCR analysis for specific genes within these elements. However, the *tet*(M) transposon, νSa encoded hyaluronidase, plasmid encoded streptomycin resistance and type V SCC*mec *encoded *ccrC *were identified in the majority of these isolates (Additional file 1: Table S4).

The presence of 5 different antimicrobial resistance elements, either integrated into the chromosome or located on plasmids, may reflect the relatively high antibiotic pressure in livestock farming. Two tetracycline resistance determinants, *tet*(M) on Tn*916 *and *tet*(K) on pS0385-1, were present in this MRSA isolate. The presence of two of these determinants may confer a higher level of resistance to tetracycline. Tetracycline resistance is most likely responsible for the selection of ST398 isolates. Therapeutic treatment of pigs with oxytetracycline and the treatment of a complete flock with prophylactic oxytetracycline are both common practices in pig farming [17]. Furthermore, a novel type V SCC*mec*, which encodes methicillin resistance, and harbors two distinct *ccrC *genes as well as a unique J1 region, was also identified. Recent reports have identified type V SCC*mec *elements in CA-MRSA ST59 and *S. pseudintermedius *that harbor two *ccrC *genes [31,32]. The *ccrC *encoded recombinase is required for the excision of SCC elements flanked by characteristic integration site sequences from the chromosome, and insertion into another chromosome. Integration site sequences were not found in the sequences between the 2 *ccrC *genes, suggesting that it is a single SCC element. The presence of two *ccrC *genes in a single element may enhance recombinase production resulting in an increase in the rate of SCC*mec *excision, since over expression of *ccrA/B *leads to increased excision of SCC*mec *elements [33]. Increased cassette chromosome recombinase C production may lead to an increased probability of SCC*mec *transfer between staphylococci. In addition, SCC*mec *in S0385 carries a putative copper resistance gene complex that is also present in the SCC element of *S. epidermidis *ATCC 1228. Copper is commonly used as a growth enhancer in pig farming [34]. Therefore, the presence of copper resistance genes may contribute to bacterial survival in this niche. The distinct genetic organization of SCC*mec *in S0385 is most likely derived from a complex series of recombination and rearrangement processes.

*S. aureus's *acquisition of DNA from other bacterial species is not very common, which is illustrated by the limited number of *S. aureus *lineages that have acquired SCC*mec *or *vanA *transposon resulting in MRSA and vancomycin-resistant *S. aureus*, respectively [35,36]. Although *S. aureus *has features that prevent foreign DNA acquisition, some strains appear to be more prone to serve as recipients of DNA transfer than others. In S0385, two potential mechanisms that may increase the transfer and acquisition of foreign DNA were identified. One factor is the absence of a type I R-M system on νSaβ. Other sequenced strains possess an R-M system on both νSa α and β islands. Restriction-modification systems have been postulated to be one of the key elements that prevent foreign DNA integration into the host genome by degradation of DNA that is not methylated by the R-M system's modification enzyme. Indeed, strains that harbor a dysfunctional type I R-M system are significantly more prone to accept foreign DNA via conjugation [37].

Another factor that may influence the acquisition of foreign DNA is the presence of three ICEs in the S0385 genome. One of these ICEs is present in a pseudo-SCC element flanked by integration site sequences and integrated into *orfX*. ICE encoded proteins form a type IV secretion-like system (T4SLS), which is a multi-protein complex that can hydrolyze the peptidoglycan layer of the cell-wall and transfer single-stranded DNA through both the cell wall and cell membrane. This DNA transfer model supports the hypothesis that ICE-encoded secretion systems are involved in conjugative DNA transport, which has been previously demonstrated for the pIP501 plasmid in gram-positive bacteria [38]. However, homologues of the transfer genes (*tra*-genes) of pIP501 could not be identified in the S0385 isolate. Alternatively, T4SLS may be involved in protein transport across the cell-wall of the bacterium. The presence of an ICE may have contributed to the acquisition of SCC*mec *by S0385. In addition, the ICE may also contribute to further transmission of SCC*mec *and also enhance the transfer of other mobile elements into new hosts. The tremendous diversity of resistance patterns observed among ST398 isolates suggests that they are able to horizontally acquire foreign DNA quite easily under antibiotic pressure [39].

The existence of *S. aureus *lineages with a broad host range, such as ST398, suggests that the bacterial genome can adapt to the host by acquiring specific (virulence) genes or altering gene expression. A recent publication regarding the bovine *S. aureus *ET3 genome suggested that the adaptation of some core genes and acquisition of SaPIbov containing genes that encode TSST-1 as well as a variant of enterotoxin C, contributed to host and tissue specificity [40]. In S0385 we identified SaPI-S0385, which harbors homologues of the *scn *and *vwb *genes, both of which encode exoproteins (Additional file 1: Table S3). SCIN, a product of *scn *encoded on φSa3, inhibits complement activation via the classical, alternative and lectin pathways by blocking both the C4b2a and C3bBb C3-convertases. It is human-specific as it does not inhibit complement activation in other species, including pigs [41]. SCIN encoded on SaPI-S0385 may demonstrate a broader host range or possibly be pig-specific, which would enable ST398 isolates to inhibit complement activation in their current animal hosts. The *vwb *homologue on SaPI-S0385 encodes a protein similar to vWbp which binds von Willebrand factor (vWf) [42]. The multifunctional vWf protein plays an important role in hemostasis as it mediates platelet adhesion and aggregation to exposed subendothelium and also binds and stabilizes coagulation factor VIII. The vWbp not only interacts with vWf, but also binds and activates prothrombin without cleaving it, which is the normal pathway for prothrombin activation [43]. The resulting complex can cleave fibrinogen and the resulting fibrin then contributes to the clotting process. Thus, vWbp contributes to clotting via two independent mechanisms. In addition, surface-bound *S. aureus *protein A also interacts with vWf. The presence of both surface bound and soluble proteins that bind vWf may increase the ability of *S. aureus *to adhere at sites of vascular damage, thereby helping the bacterium to gain entry into the host and cause infections. The vWbp encoded in the core genome shows some host-specificity, however both human and porcine serums exhibit comparable coagulation rates after the addition of vWbp [44]. The contribution of SaPI-encoded vWbp is not completely understood. However the presence of a second *vwb *gene may increase clotting activity or help broaden host specificity.

Interestingly, the MRSA ST398 lineage, which has successfully colonized livestock around the world, seems to lack a large number of well known virulence factors such as enterotoxins and other phage encoded toxins. These results have been confirmed by a recent study that genotyped 54 MRSA ST398 pig isolates using a diagnostic DNA microarray that includes virulence genes and microbial surface components that recognize adhesive matrix molecules [39]. Nevertheless, we identified several additional genes that encode for potential virulence factors such as hyaluronidase, SCIN and vWbp.

## Conclusions

The absence of virulence factors may explain why MRSA ST398 isolates have caused relatively little disease until now. However, the hypothesized enhanced ability of MRSA ST398 to acquire mobile genetic elements may also result in the uptake of mobile elements that encode virulence genes. For example, the first Panton-Valentine Leukocidin (PVL)-positive MRSA ST398 isolates have recently been reported [9,16,45]. Considering the vast and increasing animal and human reservoirs, we believe it will only be a matter of time before more of these isolates acquire mobile genetic elements that carry virulence factors which will increase virulence in the human host.

## Methods

### Bacterial strain and patient characteristics

Isolate S0385, an MRSA ST398 SCC*mec *type V, was obtained in 2006 from a blood culture of a 63 year old female patient with endocarditis of the mitral valve proven by detection of vegetations and an abscess visible on a transoesophageal echocardiogram. The patient had a history of renal transplantation and was treated with immunosuppressive therapy. Antimicrobial susceptibility testing showed that isolate S0385 was resistant to methicillin, ciprofloxacin and tetracycline. The isolate had *spa*-type t011 and was non-typeable using Pulsed Field Gel Electrophoresis with *Sma*I, as had been previously observed with MRSA ST398 [7].

### Genome sequencing and assembly

DNA from isolate S0385 was sequenced using pyrophosphate sequencing technology to 16 fold coverage by the 454 group of Roche Applied Sciences. Resulting reads were assembled into contigs and were placed into the correct order and direction using the USA300 FR3757 genome as a scaffold. All gaps between contigs were closed by conventional PCR using primers based on the sequence of flanking contigs followed by sequencing of the products. Several sequences (i.e. insertion elements and rRNA operons) were present more than once in the genome. Each copy of less than 1600 bp in size and all rRNA operons were entirely re-sequenced using conventional PCR and unique flanking primers to detect any polymorphisms. Larger sequences were not sequenced individually due to their length and the fact that only single nucleotide polymorphisms were expected.

Annotation was performed using the Annotation Engine, a prokaryotic annotation pipeline developed at the J. Craig Venter Institute (Rockville, MD, USA). A detailed description is available at http://www.jcvi.org/cms/research/projects/annotation-service/overview/. In short, the prediction of open reading frames (ORFs) and the identification of structural, tRNA, and tmRNA genes were conducted using Glimmer, tRNAscan-SE, BLAST and the Rfam database. Annotation was performed with BER.BLAST and HMMER against a variety of databases. The genome sequence and annotation of S0385 are deposited under the accession numbers [EMBL:AM990992, EMBL:AM990993, EMBL:AM990994 and EMBL:AM990995].

### Comparative genome analysis

DNA comparisons were performed using Kodon (Applied Maths, Gent, Belgium). The following *S. aureus *genomes and corresponding accession numbers were used for comparisons: USA300 FPR3757 [GenBank:CP000255], MRSA252 [GenBank:BX571856], MW2 [GenBank:BA000033], MSSA476 [GenBank:BX571857], N315 [GenBank:BA000018], NCTC8325 [GenBank:CP000253], COL [GenBank:CP000046], Mu50 [GenBank:BA000017], JH1 [GenBank:CP000736], ET3-1 [GenBank:AJ938182] and Newman [GenBank:AP009351].

### Detection of virulence and resistance genes

Seven MRSA isolates were obtained by the Faculty of Veterinary Medicine, Utrecht University, the Netherlands. All isolates were subjected to MLST, *spa*-typing and PCR-directed SCC*mec *typing as described previously [17]. The detection of the selected genes encoded by mobile genetic elements was performed by PCR. The following primer pairs were designed and used: SaPI-S0385 *int *(intSaPI_Fw 5'-TCCACTCTTTGATGAGTGCGCCA; intSaPI_Rv 5'- AGCGCAACAAGACGCATCTACA), *vwb *(vwb_Fw 5'-TGGGAGCGTTGTGTGCTTCAC; vwb_Rv 5'- CCTGTTCCGTTGTTCCCACCACC) *scn *(scn_Fw 5'- GCTATTGGTGTAGCTGCGTCGTCA; scn_Rv 5'- TGTGAAGCACACAACGCTCCCA); phageSa2 *int *(intSa2_Fw 5'-TCAAGTAACCCGTCAACTCGGAGA; intSa2_Rv 5'-TGAACCCTCTGTCAACATAGCTCGAA); phageSa6 *int *(intSa6_Fw 5'- GGCGATTTTTCTTCTTGAACCTGCGG; intSa6_Rv 5'-GGCGATTTTTCTTCTTGAACCTGCGG); ICESa1 *int *(intICE1_Fw 5'- ACGGGTTCGTGCCTCACACA; intICE1_Rv 5'-GCGTGGCAAATTAGACGTAGGGGC) ICESa2 *int *(intICE2_Fw 5'- TGCTTCATTCGTGGACGCTGAT; intICE2_Rv 5'- AAGATATGCGCCGAGGTGGAAAAA); νSaβ *hyl *(hyl_Fw 5'-TCTGCCACTGGTAAAGCTCGCA; *hyl*_Rv 5'-CTGCACCGGTGTGCCAACCT) *hsdS *(hsdS_Fw 5' GCTGGTGCATTACCTGTGACAAATGC; hsdS_Rv 5'-ATGGTGCAAAATGGGGGCAGT); *tet*(M) (tetM_Fw 5'- GACGACGGGGCTGGCAAACA; tetM_rv 5'- GCCGCCAAATCCTTTCTGGGCT); *tet*(K) (tetK_Fw 5' GCCCACCAGAAAACAAACCAAGCA; tetK_Rv 5'- AGGATCTGCTGCATTCCCTTCACT); *str *(str_Fw 5'- TGCTCTCGAGGGTTCAAGAACTAATGA; str_Rv 5'- ACACCCTTTGCTACATACGTTGAGAC).

## Authors' contributions

MJS: editing and assembly of sequencing data, annotation and bioinformatic analyses of the sequence and also writing of the manuscript. CHEB: bioinformatics analyses and critical revising of the manuscript; JAGS: conception and design of the study and critical revising of the manuscript; ACF: conception and design of the study, editing and assembly of sequencing data, annotation and bioinformatic analyses as well as editing of the manuscript. All of the authors have read and approved the final version of this manuscript.

## Supplementary Material

Additional file 1 **Supplementary tables**. Table S1 - ORFs in SCC*mec *from S0385. Table S2 - ORFs in ICESa1 from S0385. Table S3 - ORFs in SAPI from S0385. Table S4 - Detection of genes encoded by mobile genetic elements. Click here for file
