# Supplementary material for: Whole genome analysis of a livestock-associated methicillin-resistant Staphylococcus aureus ST398 isolate from a case of human endocarditis
Source: BMC Genomics. 2010 Jun 14;11:376. doi: 10.1186/1471-2164-11-376 (PMC2900268; doi:10.1186/1471-2164-11-376)
Supplement: Additional file 1 — Supplementary tables. Table S1 - ORFs in SCCmec from S0385. Table S2 - ORFs in ICESa1 from S0385. Table S3 - ORFs in SAPI from S0385. Table S4 - Detection of genes encoded by mobile genetic elements. [file 1471-2164-11-376-S1.DOC]

**Table S1: ORFs in SCC*mec* from S0385**

| **ORF** | **Protein size (aa)** | **% Identity** | **Homologous ORF** | **Homologous ORF present in^a^** | **Protein function** |
| --- | --- | --- | --- | --- | --- |
| SAPIG0028 | 101 | 98 | CZ078 | SCC*Hg* | Hypothetical protein |
| SAPIG0029 | 287 | 96 | CZ077 | SCC*Hg* | Hypothetical protein |
| SAPIG0030 | 491 | 100 | CZ076 | SCC*Hg* | Hypothetical protein |
| SAPIG0031 | 366 | 100 | CZ075 | SCC*Hg* | Hypothetical protein |
| SAPIG0032 | 123 | 94 | CZ074 | SCC*Hg* | Hypothetical protein |
| SAPIG0033 | 547 | 99 | CZ073 | SCC*Hg* | Hypothetical protein |
| SAPIG0034 | 517 | 97 | CZ072 | SCC*Hg* | Cassette chromosome recombinase C |
| SAPIG0035 | 113 | 90 | CZ070 | SCC*Hg* | Hypothetical protein |
| SAPIG0036 | 103 | 82 | CZ069 | SCC*Hg* | Hypothetical protein |
| SAPIG0037 | 168 | 90 | CZ068 | SCC*Hg* | Hypothetical protein |
| SAPIG0038 | 224 | 98 | V003a | type V SCC*mec* | Transposase for IS*431* |
| SAPIG0039 | 55 | 100 | V003b | type V SCC*mec* | HMG-CoA synthase |
| SAPIG0040 | 247 | 100 | V004 | type V SCC*mec* | Glycerophophory diester phosphodiesterase |
| SAPIG0041 | 192 | 100 | V005 | type V SCC*mec* | MaoC-like domain protein |
| SAPIG0042 | 668 | 100 | V006 | type V SCC*mec* | Penicillin-binding protein 2' |
| SAPIG0043 | 36 | 97 | V007 | type V SCC*mec* | Truncated methicillin resistance regulator |
| SAPIG0044 | 47 | 98 | V008 | type V SCC*mec* | Truncated transposase for IS*431* |
| SAPIG0045 | 142 | 100 | V009 | type V SCC*mec* | Hypothetical protein |
| SAPIG0046 | 309 | 99 | V010 | type V SCC*mec* | Hypothetical protein |
| SAPIG0047 | 662 | 100 | V011 | type V SCC*mec* | Hypothetical protein |
| SAPIG0048 | 309 | 97 | V012 | type V SCC*mec* | Hypothetical protein |
| SAPIG0049 | 122 | 66 | V013 | type V SCC*mec* | Hypothetical protein |
| SAPIG0050 | 538 | 77 | V014 | type V SCC*mec* | Hypothetical protein |
| SAPIG0051 | 559 | 95 | V015 | type V SCC*mec* | Cassette chromosome recombinase C |
| SAPIG0052 | 112 | 91 | V016 | type V SCC*mec* | Hypothetical protein |
| SAPIG0053 | 103 | 90 | V017 | type V SCC*mec* | Hypothetical protein |
| SAPIG0054 | 157 | 88 | V019 | type V SCC*mec* | Hypothetical protein |
| SAPIG0055 | 224 | - | - | SCC*mec* | Transposase for IS*431* |
| SAPIG0056 | 175 | - | - | - | Putative DNA topoisomerase III |
| SAPIG0057 | 204 | 91 | SE0058 | SCC*pbp4* | Hypothetical protein |
| SAPIG0058 | 90 | 99 | SE0059 | SCC*pbp4* | Cassette chromosome recombinase A |
| SAPIG0059 | 119 | 100 | SE0060 | SCC*pbp4* | Hypothetical protein |
| SAPIG0060 | 641 | 99 | SE0062 | SCC*pbp4* | Copper-transporting ATPase |
| SAPIG0061 | 186 | 92 | ORF7 | SCC 12263 | Truncated metallo hydrolase protein |
| SAPIG0062 | 248 | 98 | ORF7 | SCC 12263 | Truncated metallo hydrolase protein |
| SAPIG0063 | 355 | 96 | ORF6 | SCC 12263 | Coenzyme A disulfide reductase |
| SAPIG0064 | 86 | 99 | ORF5 | SCC 12263 | Hypothetical protein |
| SAPIG0065 | 243 | 99 | ORF4 | SCC 12263 | Hypothetical protein |
| SAPIG0066 | 56 | 100 | ORF3 | SCC 12263 | Partial copper ATPase |
| SAPIG0067 | 181 | 99 | ORF2 | SCC 12263 | Lipoprotein |
| SAPIG0068 | 596 | - | - | - | Hypothetical protein |
| SAPIG0069 | 217 | - | - | - | Hypothetical protein |
| SAPIG0070 | 117 | 100 | KC02a | type II SCC*mec* | Integrase |
| SAPIG0071 | 167 | 100 | KC02b | type II SCC*mec* | N- region of integrase |
| SAPIG0072 | 118 | 51 | KC03 | type II SCC*mec* | Lipoprotein |
| SAPIG0073 | 196 | 99 | KC04 | type II SCC*mec* | Hypothetical protein |
| SAPIG0074 | 348 | 98 | KC05 | type II SCC*mec* | Transfer complex protein TraG |
| SAPIG0075 | 622 | 97 | KC06 | type II SCC*mec* | Hypothetical membrane protein |
| SAPIG0076 | 452 | 99 | KC07 | type II SCC*mec* | Ftsk/spoIIIe family protein |
| SAPIG0077 | 831 | 100 | KC08 | type II SCC*mec* | Hypothetical protein |
| SAPIG0078 | 127 | 100 | KC09 | type II SCC*mec* | Hypothetical protein |
| SAPIG0079 | 86 | 80 | KC10 | type II SCC*mec* | Hypothetical protein |
| SAPIG0080 | 351 | 100 | KC11 | type II SCC*mec* | Hypothetical protein |
| SAPIG0081 | 363 | 99 | KC12 | type II SCC*mec* | Replication initiation factor family protein |
| SAPIG0082 | 100 | 100 | KC13 | type II SCC*mec* | Hypothetical protein |
| SAPIG0083 | 106 | 100 | KC14 | type II SCC*mec* | Hypothetical protein |
| SAPIG0084 | 94 | 98 | KC15 | type II SCC*mec* | Hypothetical protein |
| SAPIG0085 | 164 | - | - | - | Hypothetical protein |

^a^ GenBank database accession no.: AB037671 (SCC*Mg*, strain85/2082), AB121219 (type V SCC*mec*, strain WIS), BK001539 (SCC*pbp4*, strain ATCC12228), AB063171 (SCC 12263, strain GIFU12263), AB435014 (type II SCC*mec*, strain JCSC6826).

**Table S2: ORFs in ICESa1 from S0385**

| **ORF** | **Protein size (aa)** | **Homologous ORF in ICESa2** | **% identity** | **Homologous ORF in ICEBs1** | **% identity^a^** | **Homologous ORF in ICELm1** | **% identity^a^** |
| --- | --- | --- | --- | --- | --- | --- | --- |
| SAPIG0070 | 117 | SAPIG1848 | 61 | - |  | - |  |
| SAPIG0071 | 167 | SAPIG1849 | 49 | - |  | - |  |
| SAPIG0072 | 118 | SAPIG1850 | 41 | - |  | - |  |
| SAPIG0073 | 196 | SAPIG1851 | 51 | BSU0499 | 28 (1-100) | - |  |
| SAPIG0074 | 348 | SAPIG1852 | 62 | - |  | LIMG1204 | 37 (12-175) |
| SAPIG0075 | 622 | SAPIG1853 | 50 | BSU0496 | 25 (1-414) | LIMG1205 | 24 (35-545) |
| SAPIG0076 | 452 | SAPIG1855 | 80 | BSU0486 | 31 (9-449) | LIMG1216 | 31 (131-447) |
| SAPIG0077 | 831 | SAPIG1859 | 85 | BSU0494 | 38 (37-829) | LIMG1206 | 38 (3-830) |
| SAPIG0078 | 127 | SAPIG1860 | 61 | BSU0493 | 20 (1-127) | - |  |
| SAPIG0079 | 86 | SAPIG1861 | 63 | BSU0492 | 38 (6-60) | LIMG1207 | 31 (6-78) |
| SAPIG0080 | 351 | SAPIG1862 | 67 | BSU0491 | 30 (17-342) | LIMG1208 | 28 (33-347) |
| SAPIG0081 | 363 | SAPIG1864 | 46 | BSU0487 | 36 (9-349) | LIMG1214 | 38 (18-313) |
| SAPIG0082 | 100 | - |  | - |  | - |  |
| SAPIG0083 | 106 | SAPIG1866 | 26 | BSU0485 | 32 (4-101) | LIMG1217 | 30 (5-100) |
| SAPIG0084 | 94 | SAPIG1867 | 68 | - |  | - |  |

**^a^ Amino acid alignment coordinates of the corresponding ORF in S0385 are shown between brackets**

**Table S3: ORFs in SAPI from S0385**

| **ORF** | **Protein size (aa)** | **% Identity** | **Homologous ORF** | **Homologous ORF present in** | **Protein function** |
| --- | --- | --- | --- | --- | --- |
| SAPIG0468 | 378 | 99 | SAB0342 | SAPIbov | Integrase |
| SAPIG0469 | 261 | 99 | SAB0343 | SAPIbov | Hypothetical protein |
| SAPIG0470 | 75 | 100 | SAB0344 | SAPIbov | Hypothetical protein |
| SAPIG0471 | 86 | 100 | SAB0345 | SAPIbov | Hypothetical protein |
| SAPIG0472 | 99 | 64 | SAUSA300_0808 | SAPI5 USA300 | Hypothetical protein |
| SAPIG0473 | 787 | 83 | SAUSA300_0809 | SAPI5 USA300 | Primase |
| SAPIG0474 | 114 | 100 | SAUSA300_0810 | SAPI5 USA300 | Hypothetical protein |
| SAPIG0475 | 68 | 100 | SAUSA300_0811 | SAPI5 USA300 | Hypothetical protein |
| SAPIG0476 | 352 | 92 | SAUSA300_0812 | SAPI5 USA300 | Hypothetical protein |
| SAPIG0477 | 163 | 99 | SAUSA300_0813 | SAPI5 USA300 | Hypothetical protein |
| SAPIG0478 | 329 | - | - | - | Hypothetical protein |
| SAPIG0479 | 116 | 50 | SAUSA300_1919 | φSa3 | Stapylococcal complement inhibitor |
| SAPIG0480 | 499 | 59 | SAB0745 | ET3 | von Willebrand factor-binding protein |

**Table S**4: Detection of genes encoded by mobile genetic elements

| **Mobile genetic element** | | | | **SaPIG-S0385** | | | **ICE Sa1** | **ICE Sa2** | **phage Sa6** | **phage Sa2** | **νSaß** | | **Tn*916*** | **pS0385-1** | **pS0385-2** |
| --- | --- | --- | --- | --- | --- | --- | --- | --- | --- | --- | --- | --- | --- | --- | --- |
| **Isolate** | **Source** | ***spa* type** | **SCC*mec* type** | ***int*** | ***vwb*** | ***scn*** | ***int*** | ***int*** | ***int*** | ***int*** | ***hyl*** | ***hsdS*** | ***tet*(M)** | ***tet*(K)** | ***str*** |
| S0623 | pig | t567 | V | **-** | **-** | **-** | **-** | **-** | **-** | **+** | **+** | **-** | **+** | **-** | **+** |
| S0635 | pig | t108 | V | **-** | **-** | **-** | **-** | **-** | **-** | **-** | **+** | **-** | **+** | **+** | **-** |
| S0644 | pig | t011 | IV | **+** | **+** | **+** | **-** | **-** | **+** | **-** | **+** | **-** | **+** | **-** | **+** |
| S0655 | pig | t899 | V | **-** | **-** | **-** | **-** | **-** | **-** | **+** | **+** | **-** | **+** | **-** | **+** |
| S0654 | pig | t1939 | V | **-** | **-** | **-** | **-** | **-** | **-** | **+** | **+** | **-** | **+** | **-** | **+** |
| S0658 | pig | t899 | V | **+** | **+** | **+** | **+** | **+** | **+** | **+** | **+** | **-** | **+** | **+** | **+** |
| S0606 | pig | t567 | V | **-** | **-** | **-** | **-** | **-** | **-** | **+** | **+** | **-** | **+** | **-** | **+** |
| S0385 | human | t11 | V | **+** | **+** | **+** | **+** | **+** | **+** | **+** | **+** | **-** | **+** | **+** | **+** |
